# A Novel High Isolation 4-Port Compact MIMO Antenna with DGS for 5G Applications

**DOI:** 10.3390/mi14071309

**Published:** 2023-06-26

**Authors:** Cem Güler, Sena Esen Bayer Keskin

**Affiliations:** 1 Department of Airframe and Powerplant Maintenance, Kırklareli University, Kırklareli 39750, Turkey; 2 Electrical and Electronics Engineering Department, Kırklareli University, Kırklareli 39100, Turkey

**Keywords:** 5G, MIMO, isolation, slot, ECC, DGS

## Abstract

This paper presents the design and realization of a simple and low-profile, four-port multiple-input-multiple-output (MIMO) antenna operating in a mm-wave band supporting 5G communication technologies. As part of the design methodology, the initial stage involved the development of a conventional monopole patch antenna optimized for operation at 26 GHz, which was matched to a 50 Ω stepped feed line. Afterward, a square-shaped defected ground structure (DGS) with semi-circle slots on the edges was placed on the ground to improve the isolation, and the circular and rectangular slots were incorporated as DGSs to optimize the antenna impedance bandwidth. Etching semi-circular-shaped slots on the ground plane achieved more than 34.2 dB isolation in the 26 GHz operating band. In addition, an arrangement of four symmetrical radiating elements was positioned orthogonally to minimize the antenna’s physical size and improve the isolation. The proposed MIMO antenna’s overall dimension was 25 × 25 mm${}^{2}$, which was printed on a Rogers 5880 substrate at a width of 0.787 mm and $\varepsilon_{r}$ = 2.2. The proposed antenna covered the 5G mm-wave band with a 10 dB bandwidth ranging from 25.28–28.02 GHz, whereas the maximum gain attained for the proposed structure was 8.72 dBi. Additionally, the implementation of these slots effectively mitigated mutual coupling, resulting in reduced envelope correlation coefficient (ECC) values. Furthermore, other MIMO performance metrics, including channel capacity loss (CCL), mean effective gain (MEG), and diversity gain (DG), were analyzed for the proposed structure. The obtained results indicate its suitability for various usage areas, such as smart devices, mobile phones, and sensors in 5G applications.

## 1. Introduction

Wireless communication systems have become essential in various areas, such as health, agriculture, finance, education, the Internet of Things, media, and entertainment [1,2,3,4,5]. Mobile communication generations must be updated approximately every ten years as the demand for faster and more reliable connections grows [6]. The latest generation of wireless communication systems, 5G technology, offers several advantages over previous generations, including higher data rates, reliable connections, and reduced latency in three different usage scenarios (eMBB, uRLLC, and mMTC) [7]. In addition, 5G technology can decrease energy consumption by up to 90% [8,9]. However, the use of a single antenna in the millimeter-wave region, such as the 26 GHz band, results in signal quality degradation due to atmospheric conditions and path loss attenuation [10]. These challenges need to be addressed to ensure the optimal performance of wireless communication systems in the millimeter-wave region. MIMO technology employs spatial multiplexing techniques that enable high data transfer rates while maintaining signal quality, even in challenging environments [11,12]. Using MIMO antennas provides the high data transmission rates that 5G technology is expected to offer through spatial multiplexing [13,14,15]. Nevertheless, in MIMO antenna design, since the antenna elements share a single dielectric layer, the mutual coupling between antenna elements caused by their close proximity in MIMO systems is a challenge that needs to be addressed [16,17]. DRAs provide channel capacity, data rate, connection reliability, and high isolation in MIMO antenna systems [18,19,20]. With DRA placed on the antenna without glue, more than −15 dB isolation is obtained [21]. In another study, DRAs embedded in the antenna achieved a 7.42 dB improvement in antenna isolation [22]. However, DRAs have disadvantages, such as being sensitive to environmental effects (the presence of nearby objects and surface reflections) and requiring extra volume on the antenna. EBG structures are commonly used in 5G millimeter-wave applications to increase isolation between MIMO antenna elements by restricting the movement of surface current waves [23,24]. Studies have reported EBG structures providing isolation values of over 25 dB [25], with other studies achieving 23 dB isolation values between radiating antennas [26]. However, the complexity and production difficulties of EBG designs, low bandwidth, and low gain values have limited the use of this method. Decoupling networks (DNs) have been proposed to reduce mutual coupling between antenna elements, offering enhanced gain and reduced interference [27]. However, they also have significant drawbacks, requiring more space between radiating elements and potentially increasing power losses. In MIMO antenna systems, DNs have been shown to reduce mutual interaction between elements, achieving an isolation level of 20 dB [28]. Nonetheless, ensuring the stability of the DN is crucial, as any instability can severely impact the antenna’s performance and bandwidth. Using parasitic elements (PEs) can improve isolation levels [29]. An asymptotic structure to reduce the connection between antenna elements achieves an isolation value of 16.4 dB [30]. It is stated that using C-shaped parasitic elements between MIMO antenna elements improves mutual coupling by 8.58 dB to reduce the connection between antennas [31]. Although PEs successfully improve the isolation level, adding PEs between the elements causes a shift in the antenna’s frequency and requires redesigning the antenna. Therefore, using different parasitic elements for antennas operating at different frequencies is impractical. Neutralization lines are preferred in MIMO antenna designs due to their ability to facilitate easy impedance matching. Using NL in a MIMO antenna designed using characteristic mode analysis results in an isolation value of 16 dB [32]. NL used in two- and four-port MIMO antennas achieve an isolation value of over 22 dB and 23 dB, respectively [33]. The length of the NL, which is preferred at low frequencies, depends on the antenna frequency, and as the bandwidth increases, the length of the line also increases, causing additional cost problems. In addition, it is crucial to install NL correctly. More installation is needed to maintain the efficiency of the lines. Additionally, hybrid isolation development techniques emerge with the combined use of these methods. Various hybrid methods exist, such as the use of EBG and DGS together [34], L-shaped stubs, defective ground, and chip resistors used together [35], the use of the DGS method on the ground plane and epsilon-and-mu-near-zero-based metasurface superstrate [36], and the use of slot and parasitic element structures [37]. Moreover, various techniques have been employed to enhance the isolation levels in MIMO antennas by mitigating mutual coupling effects, such as the decoupling network [38], the integration of vias [39], a zigzag-shaped slotted structure in the ground plane [40], a partially reflecting surface [41], a partial ground surface [42], a partial ground surface combined with metasurface [43], a slot in the ground plane [44,45,46,47,48,49,50,51], decoupling branches [52], and a stub in the ground plane [53]. The techniques for enhancing isolation used in prior research are known to be expensive and involve several processing steps. As antenna dimensions decrease in the millimeter-wave band, producing complex designs becomes increasingly challenging. Numerous research studies have focused on the connected ground configuration in MIMO antennas [54,55,56,57,58]. However, achieving high isolation between antenna elements in this configuration can be difficult, resulting in reduced system capacity and overall performance. To address these difficulties, this article introduces a novel four-port MIMO antenna design with a separate ground configuration that operates in the millimeter-wave band and exhibits a low profile, small size, wide bandwidth, and high isolation value compared to the studies in the literature. The incorporation of two rectangular slots in the ground plane, positioned immediately behind the junction of the transmission line and the patch as well as the circular slot integrated in the ground plane, facilitates coverage of the frequency band spectrum designated for 5G millimeter-wave applications by optimizing the center frequency within the bandwidth. In addition, the proposed design features orthogonally placed radiating patches that minimize the physical size of the antenna and enhance isolation. In addition, a semicircular DGS is incorporated at the edges of the ground plane to further improve isolation between antenna elements. This is due to the fact that the curved geometry of the semi-circular slots helps to redirect the coupling effect away from the antenna elements and reduce the coupling between adjacent antenna elements, resulting in a stronger isolation between the ports. Overall, the proposed design employs the defected ground structure to reduce the envelope correlation coefficient (ECC), a critical parameter for MIMO antennas. The novelty of the proposed antenna design lies in the unique combination of a compact and simple design, easy fabrication of the microstrip antenna with DGS, cost-effectiveness, and its suitability as a good candidate for use in 5G systems with improved isolation levels.

## 2. Proposed Antenna Design

The usage of MIMO antennas in mm-wave applications is particularly valuable due to their low cost, low profile, and compact size. The appropriate design of four-port MIMO antennas can result in strong isolation levels and optimal radiation characteristics, which are crucial for achieving high-performance wireless communication systems. The proposed antenna was designed with a stepped line feed, rectangular and circular slots implemented as defected ground structures for achieving wider bandwidth, as well as optimal radiation characteristics. Moreover, it has been determined that the optimal radiation characteristics of the antenna can be achieved by incorporating semi-circular slots at the edges of the ground surface of the antenna elements, and by employing an orthogonal arrangement of the four antenna elements to achieve a strong isolation of at least 34.2 dB.

### 2.1. Design of the Unit Cell

**Figure 1:**
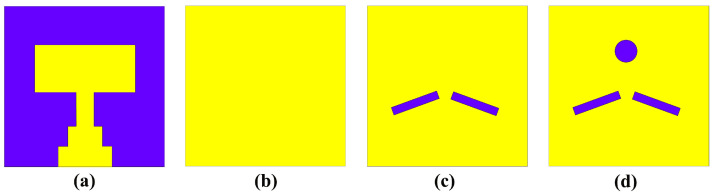
Design steps of the proposed unit cell. (**a**) Front view of the unit cell, (**b**) back view of the unit cell—step I, (**c**) back view of the unit cell—step II, (**d**) back view of the unit cell—step III.

The unit cell of the proposed antenna comprises DGS and an antenna radiator, developed in three steps, incorporating various geometrical shapes, such as rectangular and circular slots, and the design steps of the unit cell illustrated in Figure 1. Figure 1a demonstrates the front view (radiating element) of the unit cell, while Figure 1b–d show the back view design steps (ground plane) of the unit cell. In Step I, the conventional microstrip feed line is optimized with a stepped feed line structure, as illustrated in Figure 1a. This modification is aimed at enhancing the antenna’s return loss and bandwidth characteristics. The stepped microstrip feed line design enables improved impedance matching and reduced signal reflection, thereby resulting in enhanced antenna performance. The ground surface of the unit cell in Step I is shown in Figure 1b. In Step II, two rectangular-shaped slots are etched into the ground plane, positioned directly behind the junction of the transmission line and the patch surface, creating DGS, as illustrated in Figure 1c. This modification effectively eliminates surface waves and results in improved performance of the antenna. The design with DGS enables a return loss of −30.93 dB to be achieved, as shown in Figure 1c. In Step III, the proposed ground plane design of the unit cell is presented, which includes the addition of a circular slot at the full-back alignment of the patch’s top, as shown in Figure 1d. This modification enables the antenna to achieve a return loss of −38.65 dB, a bandwidth of 25.3 to 27.6 GHz, and a center frequency of 26.26 GHz. By optimizing the center frequency within the bandwidth, the circular structure at the top of the patch helps to cover the frequency band spectrum designated for 5G mm-wave applications. In addition, to design the proposed antenna, Equations (1)–(7) from [59] are used.

The width (*P*${}_{W}$) and the length ($P_{L}$) of the patch are determined by applying the following equations.

(1) $$\begin{array}{c} P_{W}=\frac{c}{2f_{r}}\sqrt{\frac{2}{\varepsilon_{r}+1}} \end{array}$$

Here, *c* represents the speed of light (3 × 10${}^{8}$ m/s), $\varepsilon_{r}$ denotes the permittivity of the substrate, and *f* ${}_{r}$ refers to the resonance frequency.

(2) $$\begin{array}{c} P_{L}=L_{eff}-2\Delta L \end{array}$$

$L_{eff}$ and ΔL can be calculated using the following equations:

(3) $$\begin{array}{c} L_{eff}=\frac{c}{2f_{r}\sqrt{\varepsilon_{reff}}} \end{array}$$

(4) $$\begin{array}{c} \Delta L=0.412h\frac{\left(\varepsilon_{reff}+0.3\right)\left(\frac{P_{W}}{h}+0.264\right)}{\left(\varepsilon_{reff}-0.258\right)\left(\frac{P_{W}}{h}+0.8\right)} \end{array}$$

Here, *h* represents the height of the substrate and the effective dielectric constant $\varepsilon_{reff}$ is given by:

(5) $$\begin{array}{c} \varepsilon_{reff}=\frac{\varepsilon_{r}+1}{2}+\frac{\varepsilon_{r}-1}{2}{\left(1+12\frac{h}{P_{W}}\right)}^{-0.5},\frac{P_{W}}{h}>1 \end{array}$$

Then, the ground width ($D_{W}$) and ground length ($D_{L}$) are calculated using the following equations:

(6) $$\begin{array}{c} D_{W}=6h+P_{W} \end{array}$$

(7) $$\begin{array}{c} D_{L}=6h+P_{L} \end{array}$$

**Figure 2:**
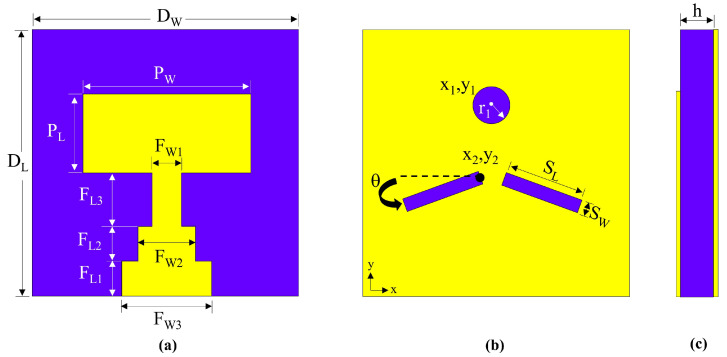
Design of the proposed unit cell of the MIMO antenna. (**a**) Front view, (**b**) back view, and (**c**) side view of the unit cell.

The unit cell of the proposed antenna results in the specific form illustrated in Figure 2. Figure 2a demonstrates the front view (radiating element), Figure 2b shows the back view (ground plane), and Figure 2c displays the side view of the unit cell.

**Figure 3:**
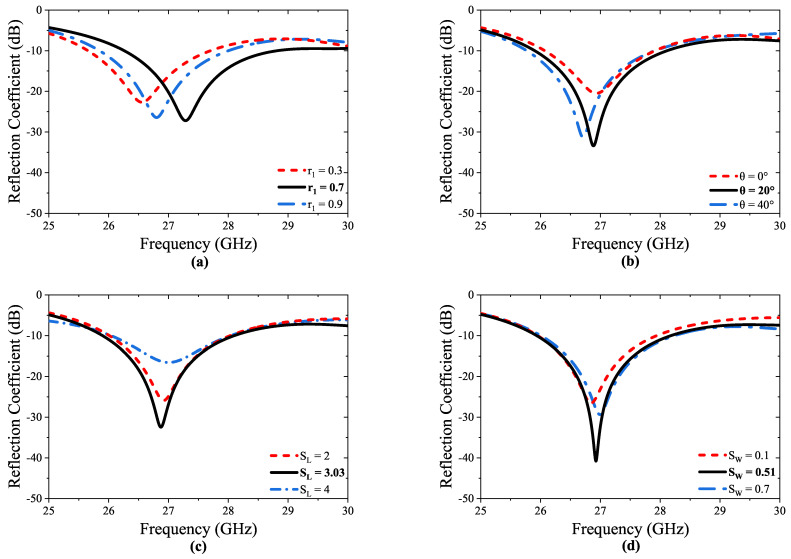
Parametric analysis of the unit cell: (**a**) $r_{1}$, (**b**) θ, (**c**) $S_{L}$, and (**d**) $S_{W}$.

The utilization of the DGS technique can significantly enhance the bandwidth of the unit cell in the antenna design, thereby improving the overall performance across a wider range of operating frequencies. Consequently, the introduction of a circular slot at the center of the ground plane, along with two rectangular slots positioned immediately behind the junction of the transmission line, facilitates the creation of the DGS. These slots indicate a defect in the ground plane, which affects the surface current distribution on the ground plane and, hence, the impedance and bandwidth of the antenna. Therefore, the diameters of the circular slot ($r_{1}$) and placement angle of rectangles (θ), as well as the length ($S_{L}$) and width ($S_{W}$) of the rectangles, are optimized to improve the bandwidth, as shown in Figure 3. The optimized geometric dimensions of the proposed unit cell are presented in Table 1.

**Figure 4:**
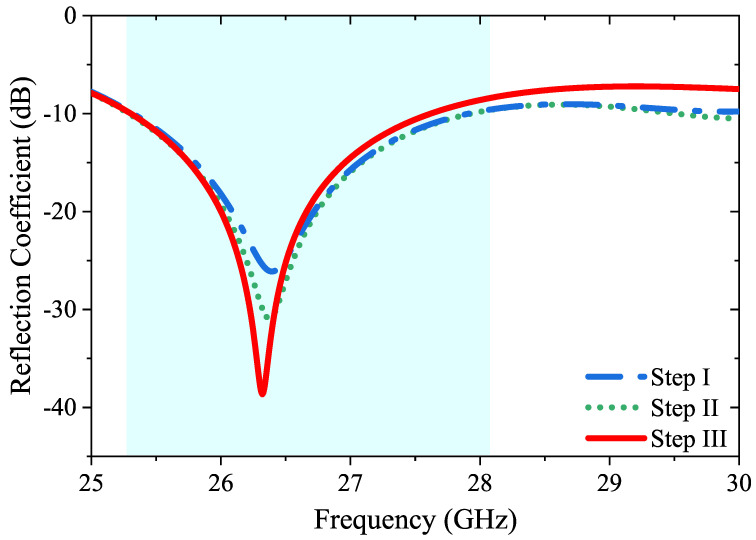
Reflection coefficient values for each antenna design step (Step I, Step II, and Step III).

The reflection coefficients ($S_{11}$) corresponding to following design steps (Step I, Step II, and Step III) are depicted in Figure 4.The major resonance frequency of the MIMO system is 26.3 GHz, and the return loss values ranged from −26.1 to −38.6 dB. Here, the light blue background representation in the following graphs serves to highlight the specific 10 dB operating band of the 4-port MIMO antenna.

### 2.2. Four-Port MIMO Antenna for 26 GHz 5G Application

**Figure 5:**
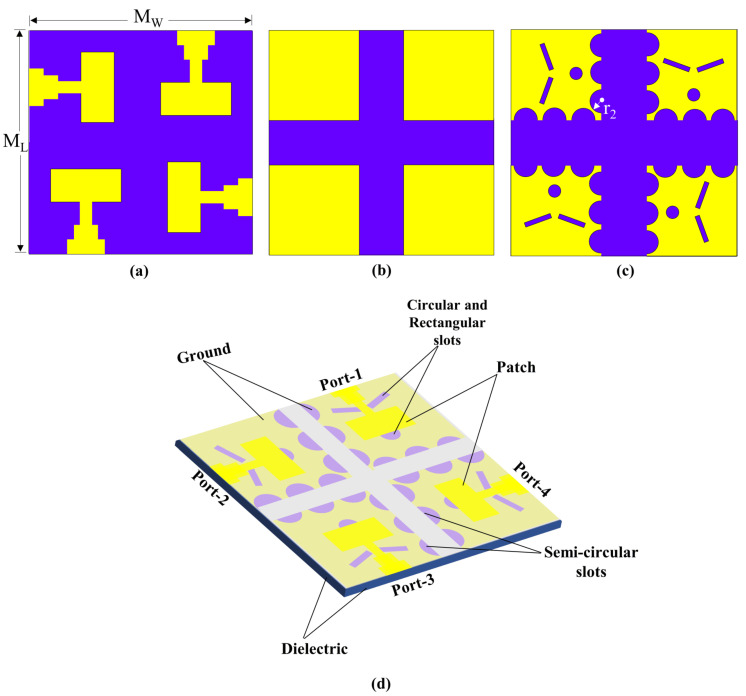
Design evolution of the proposed 4-port MIMO antenna, (**a**) top layer ($M_{L}$ = $M_{W}$ = 25 mm), (**b**) bottom layer—Step I, (**c**) bottom layer—proposed, and (**d**) 3D visualization of the physical structure of the proposed 4-port MIMO antenna.

**Figure 6:**
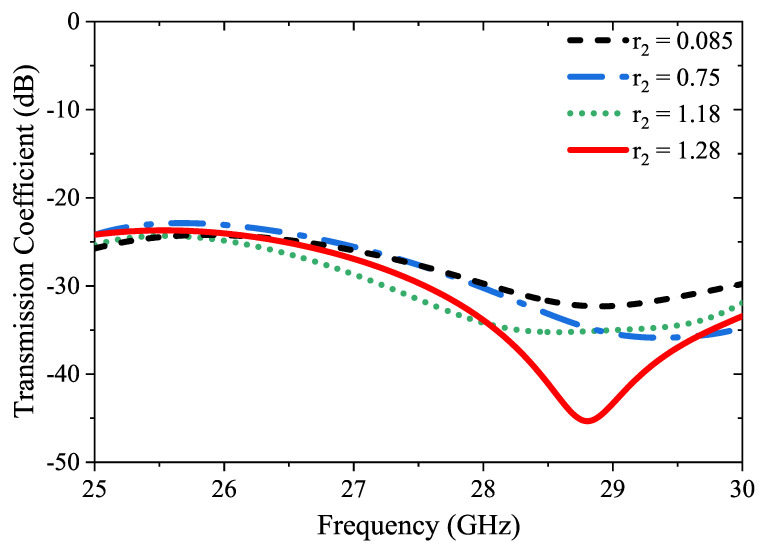
Parametric analysis of the diameter ($r_{2}$) of the semicircular slots.

**Figure 7:**
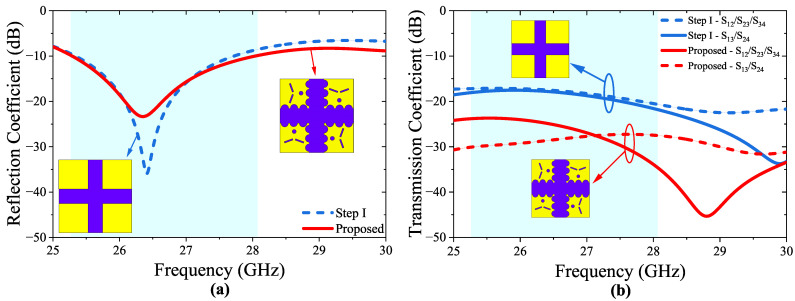
Simulated S-parameter plots, (**a**) Reflection coefficients, (**b**) Transmission coefficients of the proposed 4-port MIMO antenna.

The developmental stages and a comprehensive 3D visualization of the physical structure of the four-port MIMO antenna are presented in Figure 5. The DGS can also help to reduce the surface waves that propagate along the ground plane, which can improve the radiation efficiency of the antenna. Therefore, by introducing semi-circular slots at the edges of the ground surface as shown in Figure 5c, the geometry of the ground plane is effectively modified, which can affect the coupling between the antenna elements. Hence, the diameter ($r_{2}$) of the semicircular slots is optimized to achieve the desired level of isolation between the antenna elements, as depicted in Figure 6. It is important to note that the maximum $r_{2}$ value that can be used to ensure that the semicircular slots do not overlap and maintain their circular shape is 1.28 mm. The curved geometry of the semi-circles helps to redirect the coupling effect away from the antenna elements, reducing the amount of energy that is coupled between them. The size, shape, and placement of the semi-circle slots can be optimized to achieve the desired level of isolation between the antenna elements. In this sense, the incorporation of semi-circular slots at the edges of the ground surface of the antenna elements has been observed to enhance the isolation values of the antenna, as depicted in Figure 7a.

The proposed MIMO antenna is fabricated on an RT/duroid 5880 substrate with a dielectric constant of $\varepsilon_{r}$ = 2.2 and a loss tangent (tanδ) of 0.009, and the overall dimension of the substrate is 25 × 25 × 0.787 mm${}^{3}$. The computer simulation tool, i.e., CST Microwave Studio, was employed for designing, simulating, and optimizing the proposed antenna.

As depicted in Figure 7, it can be observed that the incorporation of semi-circular slots along the edges of the ground surface of the antenna elements resulted in a noteworthy improvement of isolation level of 13.6 dB. This is due to the fact that the semi-circular slots disturb the flow of the surface current distributions by diverting the conducting path that helps to reduce the coupling between antenna elements, resulting in a stronger isolation between the ports.

## 3. Results and Discussion

**Figure 8:**
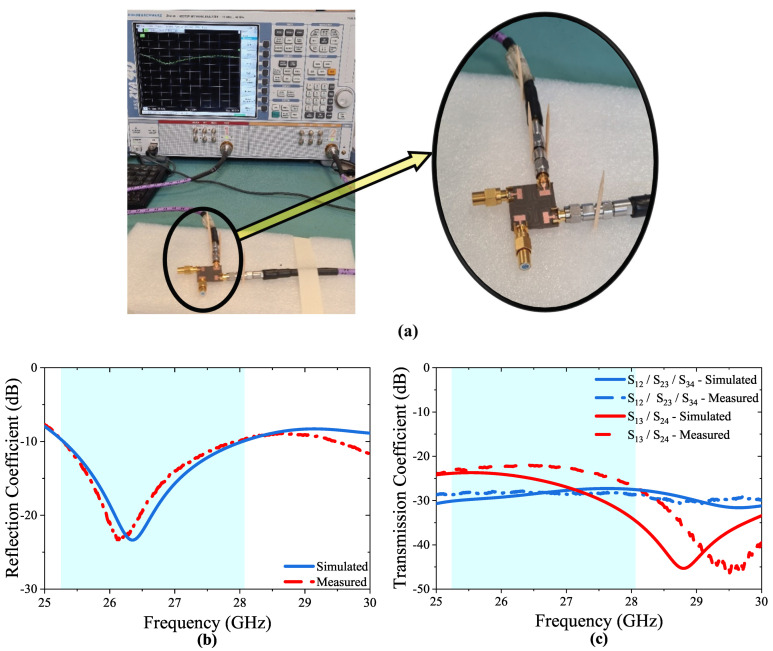
(**a**) Measurement setup, (**b**) measured and simulated $S_{11}$ denotes the lowest $S_{11}$ of −23.5 dB between 25.28 and 28.02 GHz, (**c**) measured transmission coefficients are obtained as minimum −29.4 dB for $S_{12}$, $S_{13}$ and −23.2 dB for $S_{13}$ and $S_{24}$ values.

The proposed 4-port MIMO antenna was fabricated and measurements were tested using a network analyzer, as shown in Figure 8a. The design was simulated using Microwave Studio Suite CST. A comparison between the measured and simulated reflection/transmission coefficients is presented in Figure 8b,c. The slight difference between the simulated and the measured results may have occurred due to the fabrication tolerances, SMA connector characteristics, and losses associated with coaxial cables. However, the observed differences are within an acceptable range, and the overall trends and characteristics of the antenna were still captured accurately by the simulation.

**Figure 9:**
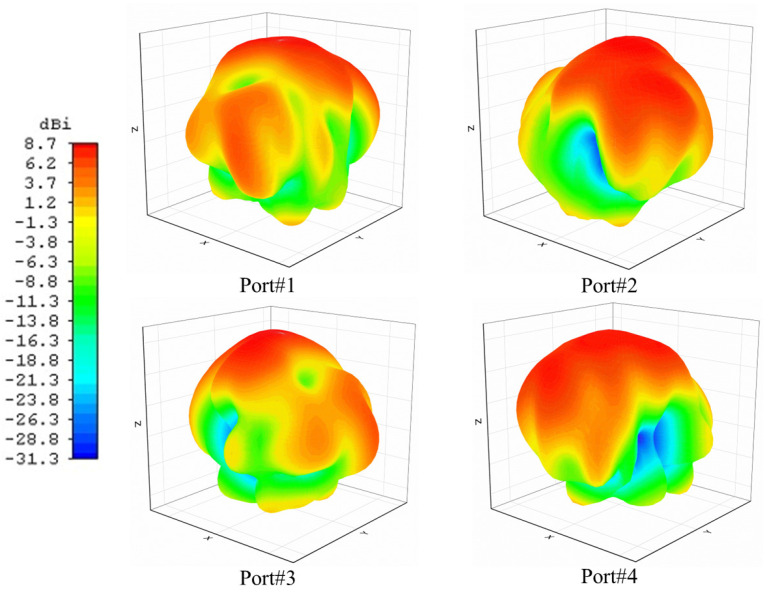
The 3D directivity gain results of each antenna element in the presented MIMO antenna.

Figure 9 illustrates the 3D radiation patterns of the proposed 4-port MIMO Antenna. As depicted in Figure 8a simulated peak gain within the desired frequency band is 8.72 dBi.

**Figure 10:**
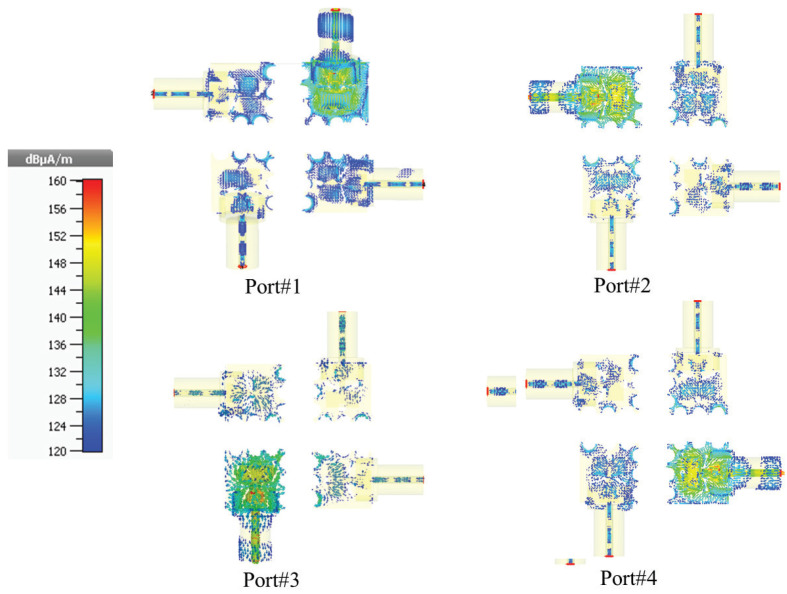
The surface current distribution of each antenna element.

Additionally, Figure 10 displays the surface current distribution plots for each antenna port, which show concentrated current distributions at the patch edges and slots drilled in the ground plane. The observation that the current distribution is higher on the patch surface where the circular and rectangular slots as DGS are located indicates that these slots have facilitated the propagation of electromagnetic waves, resulting in an improved bandwidth of the four-port MIMO antenna. Each element has a maximum surface current amplitude of 160 µA/m at a resonant frequency of 26.26 GHz In addition, the use of DGS, in the form of semi-circles placed at the edge of the ground plane, contributed to the reduction of mutual coupling between the four orthogonally placed antenna elements, as observed in Figure 10.

**Figure 11:**
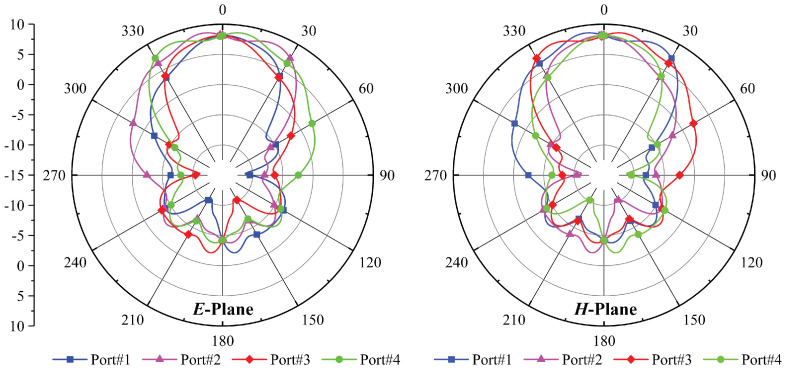
The E-plane and H-plane graphs superimposed for each port.

The E- and H-planes are simulated at a 26.26 GHz center frequency, and Figure 11 shows the Phi = 0° H-plane and Phi = 90° E-plane for each antenna port. Both antennas have a wide edge radiation pattern with low side lobes in the E-plane. For port 1, the half-power beamwidth (HPBW) is 73.9° at Phi = 0° and 46.5° at Phi = 90°. For port 2, the HPBW is 46.6° at Phi = 0° and 73.9° at Phi = 90°. For port 3, the HPBW is 73.9° at Phi = 0° and 46.6° at Phi = 90°. For port 4, the HPBW is 46.5° at Phi = 0° and 73.9° at Phi = 90°. As a result, the maximum radiation pattern is a parameter that is taken into consideration when evaluating the overall performance of the MIMO antenna.

The envelope correlation coefficient (ECC), diversity gain (DG), and total active reflection coefficient (TARC) are metrics that provide crucial information about the antenna’s ability to provide stable, high-quality, and diverse signals, thereby ensuring optimal system performance. Although MIMO antenna systems can theoretically increase a system’s capacity, the system’s performance can be negatively impacted if the signals received at different antenna elements are correlated [60]. ECC measures the degree of independence between the radiation patterns of two antennas. The theoretical assumption is that the ECC value should be zero in an ideal scenario, where the antennas can radiate independently of each other. However, in practice, due to various losses, an ECC value of <0.5 is considered acceptable. This value indicates the little interaction between the antennas and determines how well the performance is. ECC can be expressed using the S parameters given in Equation (8) and the far-field given Equation (9) for any MIMO antenna system [61]. In this study, the resulting ECC value of <0.0005 indicates a high degree of independence between the radiation patterns of the antennas and good performance, implying that the antennas are radiating independently of each other with very little interaction.

(8) $$\begin{array}{c} \mathrm{ECC}=\frac{{\left|S_{ii}^{*}S_{ij}+S_{ji}^{*}S_{jj}\right|}^{2}}{\left(1-{\left|S_{ii}\right|}^{2}-{\left|S_{ji}\right|}^{2}\right)\left(1-{\left|S_{jj}\right|}^{2}-{\left|S_{ij}\right|}^{2}\right)} \end{array}$$

Here, i,j = 1, 2, 3, 4, and “∗” is the complex conjugate of the S-parameter.

(9) $$\begin{array}{c} \mathrm{ECC}=\frac{{\left|\int \int 4\pi \left[F_{i}\left(\theta ,\phi \right)\right]•\left[F_{j}\left(\theta ,\phi \right)\right]d\Omega \right|}^{2}}{\int \int 4\pi {\left|F_{i}\left(\theta ,\phi \right)\right|}^{2}d\Omega \int \int 4\pi {\left|F_{j}\left(\theta ,\phi \right)\right|}^{2}d\Omega } \end{array}$$

where “•” denotes the Hermitian product, “Ω” is the solid angle, $F_{i}\left(\theta ,\phi \right)$ and $F_{j}\left(\theta ,\phi \right)$ represent the 3D radiation pattern fields with excitation at ports “*i*” and “*j*”, respectively. Evaluating the adequacy of MIMO features involves considering the diversity gain (DG) as another key component. Optimizing the diversity gain is crucial for achieving a high-performance MIMO antenna system. If the envelope correlation coefficient (ECC) exceeds 0.5, the system fails to provide the diversity gain, potentially degrading the system’s performance. A value of |ECC| ≤ 0.3 is considered sufficient to achieve the diversity gain [62]. The DG is derived from ECC using Equation (10) and measures the ability of antennas to reduce fading and improve the quality of the received signal in a multipath environment. The aim is to create an environment where the signals received by each antenna are as different as possible so that the receiver can combine them to improve the overall signal quality. The DG is a crucial factor in evaluating the performance of MIMO antennas as it is closely related to their ability to reduce fading and improve signal quality. Higher data rates and better system performances can be achieved by optimizing the diversity gain using Equation (10) to ensure a high diversity gain, typically around 10 dB [63].

(10) $$\begin{array}{c} \mathrm{DG}=10\sqrt{1-{\left|\mathrm{ECC}\right|}^{2}} \end{array}$$

**Figure 12:**
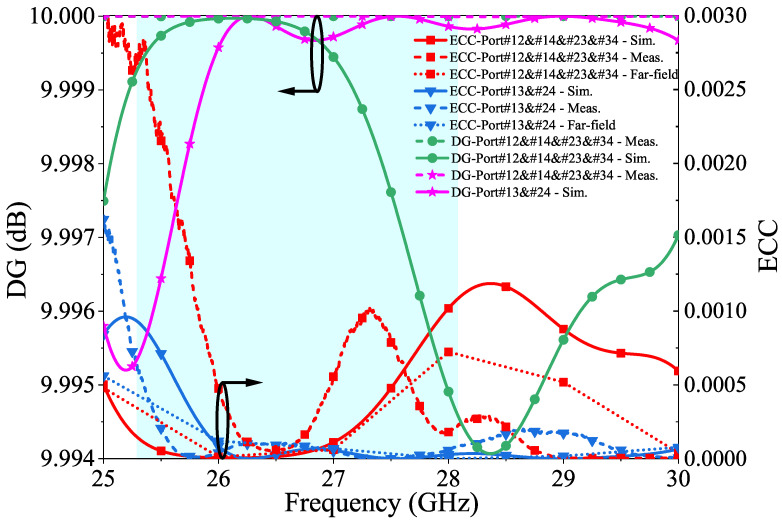
The ECC and DG measured and simulated results of the proposed 4-port MIMO antenna.

Figure 12 presents the results of the measured and simulated ECC and DG values for the proposed multiple-input multiple-output (MIMO) antenna. In this graph, black circles and arrows are used as visual cues to indicate the graphs that correspond to either the ECC or DG axis.

**Figure 13:**
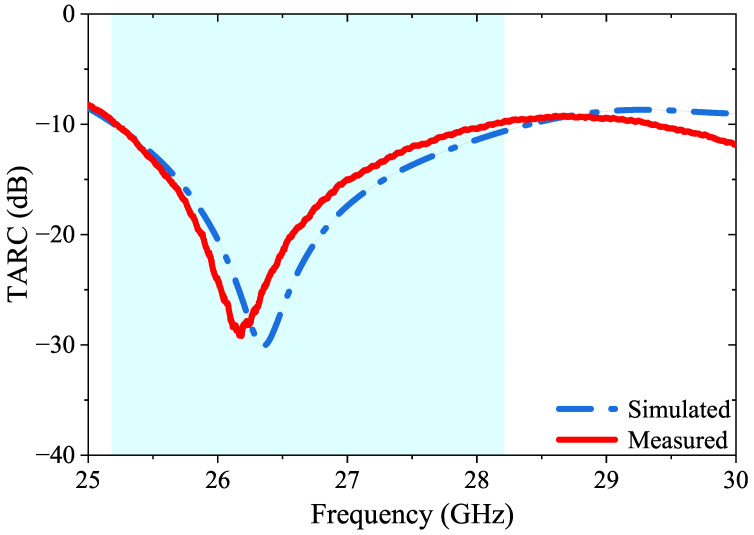
TARC measured and simulated results of the proposed 4-port MIMO antenna.

The total active reflection coefficient (TARC) is an important parameter to consider when designing a MIMO communication system. TARC is calculated by taking the ratio of the square root of the total reflected power to the total power in the system, and is estimated by using arbitrary signal combinations and measuring excessive couplings between antenna ports. It is crucial that the TARC value does not exceed 0 dB, as this could lead to distorted received results and erroneous data transmission. Figure 13 shows the simulated and measured TARC values for the proposed four-port MIMO antenna; they have been evaluated to be below 10 dB for most of the frequency range.

(11) $$\begin{array}{c} \mathrm{TARC}=-\sqrt{\frac{{\left(S_{11}+S_{12}\right)}^{2}+{\left(S_{21}+S_{22}\right)}^{2}}{2}} \end{array}$$

The voltage standing wave ratio (VSWR) is an essential metric that indicates the degree of impedance matching between the antenna and transmission line. It is defined as the ratio of the amplitudes of the forward and reflected waves along the transmission line. When the VSWR value is equal to 1, it signifies that all power is transferred to the antenna with no reflection, thereby ensuring optimal antenna performance. The simulated VSWR value of the antenna is 1.2; indicating that the impedance of the antenna matches the transmission line’s impedance, allowing maximum power to be transferred from the source to the antenna.

**Figure 14:**
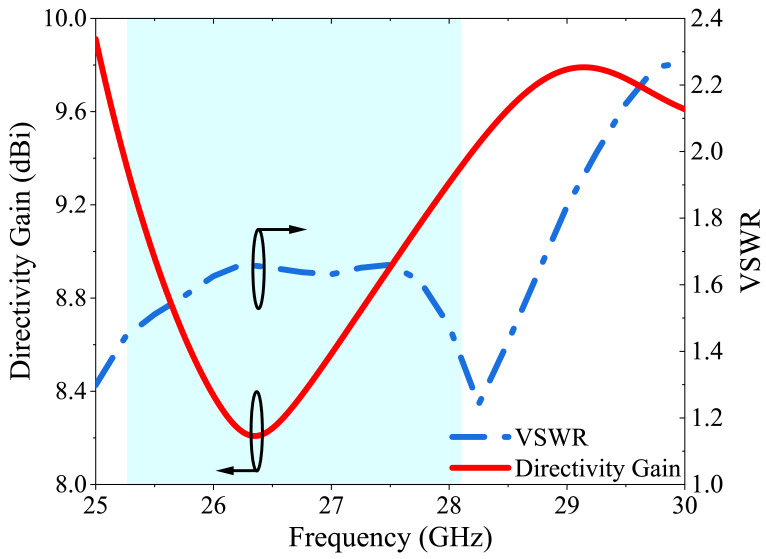
VSWR and DG results of the proposed 4-port MIMO antenna.

Another critical metric in the antenna design is directivity gain, which is the ratio of the radiation intensity of an antenna in a particular direction to the average radiation intensity of the antenna over all directions. Antennas with higher directivity gains are more efficient, allowing for better transmission of signals over longer distances or reception of weaker signals. Therefore, the directivity gain is a key factor to consider when designing and optimizing antennas for various applications, particularly in wireless communication systems. Figure 14 depicts the VSWR and DG of the four-port MIMO antenna.

Table 2 presents a comparison of the proposed MIMO antenna with the recently published literature in terms of the method used for isolation, the operating frequency of the system, the number of ports in the MIMO antenna, the size of the MIMO antenna, the realized gain, the isolation levels between antenna elements, and the effective channel capacity. It can be seen from the table that the antenna suggested in this manuscript has the lowest ECC values, excluding the designs investigated in [44,45,46,47,48,49]. Although the ECC values provided in [44,45,46,47,48,49] are lower than those of the proposed antenna in the manuscript, Ref. [44] has lower max. gain values and min. isolation values, and Ref. [49] has a higher volume antenna while having a max. gain value lower than the proposed antenna. Although the max. gain and min. isolation values of Ref. [41] are higher compared to the proposed antenna, it is observed that Ref. [41] has a complex design and a high volume structure, and the ECC value is lower than the proposed study. Furthermore, despite Ref. [49] exhibiting lower min. isolation and ECC values compared to the proposed antenna, Ref. [49] pertains to a high volume antenna, which results in a lower max. gain value compared to the proposed antenna.

## 4. Conclusions

In recent years, 5G technology has gained critical significance as a communication service. This is largely due to its ability to provide higher multi-Gbps peak data speeds, ultra-low latency, increased network capacity, heightened availability, greater reliability, and a more uniform user experience for a larger number of users. Moreover, there is a growing trend towards the development of lighter and thinner portable gadgets that demand powerful processing capabilities. This manuscript presents a novel, compact, and low-profile 4-port MIMO antenna design for millimeter-wave applications operating at 26 GHz. The antenna has dimensions of 25 × 25 × 0.787 mm${}^{3}$ and was built on a relatively small, low-cost substrate of RT/duroid 5880. It is suitable for use over a wide frequency band of 25.28–28.02 GHz, achieving a minimum isolation value of 23.2 dB and a directivity gain of 8.72 dBi. MIMO metrics, such as ECC, DG, and CCL were simulated and calculated from the measured data to verify the diversity performance of the proposed antenna and demonstrate its superior characteristics. The proposed MIMO antenna is distinct from other reported works in the literature with its wide operating bandwidth, high isolation, low ECC, design features, compact size, and low cost. Due to these characteristics, it has great potential for use in a wide range of 5G applications.

## Figures and Tables

**Table 1:** Optimized geometric dimensions of the proposed unit cell of the MIMO antenna.

Parameter	Value (mm)	Parameter	Value (mm)	Parameter	Value (mm)
$P_{W}$	6.301	$F_{L1}$ = $F_{L2}$	1.31	$r_{1}$	0.7
$P_{L}$	2.99	$F_{L3}$	2.07	$r_{2}$	1.28
$F_{W1}$	1.05	$x_{1}$, $y_{1}$	(0.0, 2.0)	$S_{W}$	3.03
$F_{W2}$	2.06	$x_{2}$, $y_{2}$	(−0.3, 0.71)	$S_{L}$	0.51
$F_{W3}$	3.21	$D_{W}$ = $D_{L}$	10	θ	20${}^{\circ }$

**Table 2:** Comparison of this study with other studies in terms of bandwidth, isolation, directivity gain, and ECC.

Ref.	Method Used for Isolation	Operating Frequency (GHz)	Design Complexity	Size ($\lambda_{0}$ × $\lambda_{0}$ × $\lambda_{0}$)	Min. Isolation (dB)	Max. Gain, (dBi)	ECC
[40]	DGS	25.5–29.6	High	2.8 × 3.27 × 0.071	17	8.3	<0.01
[41]	PRS	25–33	High	1.58 × 1.58 × 0.7	25	14.1	<0.008
[42]	PGS	3–13.5	Low	0.73 × 0.73 × 0.279	15	3.5	<0.4
[43]	PGS + MS	3.11–7.67	High	0.58 × 0.58 × 0.02	15.5	8.3	<0.004
[44]	DGS	24–34, 37–41.5	Low	0.56 × 0.19 × 0.02	20	11	<0.005
[45]	DGS	27–29	Low	2.21 × 1.4 × 0.069	17	7.8	<0.001
[46]	DGS	24.647–28.182	Low	2.65 × 2.47 × 0.044	22	6.22	<0.05
[47]	DGS	23.3–32.1	Low	1.44 × 1.44 × 0.045	15	<10	<0.03
[48]	DGS	27.2–29.2	Low	2.8 × 2.8 × 0.148	29	<7.1	<0.0005
[49]	O-DGS	25–50	Low	3.33 × 3.33 × 0.023	20	-	<0.005
[50]	DGS	24.8–27.6	Low	4.13 × 5.54 × 0.508	20	9	<0.002
[51]	Orientation	2.42–2.48	Low	7.93 × 12.26 × 0.13	20	5.37	<0.013
[52]	DB	2.9–10.86	High	1.3 × 1.3 × 0.002	22	4	<0.01
[53]	Stub	2–10.6	Low	0.82 × 0.82 × 0.029	17	3	<0.003
This Work	DGS	25.28–28.02	Low	2.21 × 2.21 × 0.069	23.2	8.72	<0.0015

## Data Availability

Not applicable.

## Abbreviations

The following abbreviations are used in this manuscript:

DGS	defected ground structure
PRS	partially reflecting surface
DPE	diagonal parasitic element
PGS	partial ground surface
MS	metasurface
O-DGS	orientation DGS
DB	decoupling branch
DG	diversity gain
